# Beyond the Learning Curve of Robot-Assisted Navigation Spine Surgery: Refinement of Outcomes With Extended Experience

**DOI:** 10.7759/cureus.69007

**Published:** 2024-09-09

**Authors:** Carlo A Benech, Rosa Perez, Isabella Lucyk, Brandon S Bucklen

**Affiliations:** 1 Department of Neurology and Clinical Neurophysiology, Fornaca Clinic, Turin, ITA; 2 Research, Globus Medical Inc., Audubon, USA

**Keywords:** continued refinement, extended experience, learning curve, robotic assisted navigation, spine surgery

## Abstract

Objective

This study assessed whether robotic-assisted navigation (RAN) spine surgery outcomes, including operative time and pedicle screw accuracy, continue to improve with extended experience beyond 200 cases.

Methods

This is a retrospective review of 60 patients who underwent lumbosacral transforaminal interbody fusion using RAN. Patients were segmented into three groups of 20 consecutive cases each. The first group represented a surgical performance baseline leading up to the investigating surgeon’s 200th RAN case. The subsequent two groups were selected beyond the 200th case with an average of 15 cases between groups. Pedicle screw accuracy and intraoperative outcomes were assessed. Statistical results were significant if p<0.05.

Results

Measures of surgical efficiency significantly improved beyond the investigating surgeon’s 200th RAN case. As case number increased, the following parameters significantly decreased: registration time (group 1: 16.9±6.5, group 2: 12.9±3.0, group 3: 8.7±1.6 minutes; p<0.05), screw insertion time (group 1: 14.9±3.5, group 2: 10.9±2.0, group 3: 8.4±2.7 minutes; p<0.05), and total operative time significantly decreased from group 1 (175.9±58.2 minutes) to group 2 (135.8±23.9 minutes) (p=0.013) with a non-significant decrease to group 3 (121.5±32.3 minutes). Accuracy (Grade = A) significantly increased across groups (group 1: 87%, group 2: 94%, group 3: 98%; p=0.024). Group 1 had the highest misplacement rate of 3.7% (4/108 screws). The overall misplacement rate was 1.4% (4/290 screws) (Grade C-E). There was a higher rate of lateral screw misplacement compared to medial misplacement.

Conclusion

Even with a small number of initial cases, RAN spine surgery can consistently be performed with high accuracy and acceptable intraoperative outcomes. However, this study demonstrated refined outcomes with extended robotic experience.

## Introduction

During the last several decades, image guidance (IG) and robotic-assisted navigation (RAN) systems have become increasingly used in spinal surgery [1]. Both are designed to assist surgeons by enabling real-time surgical navigation and robotic guidance using radiological patient images. Identified benefits have included increased accuracy [2-6], reduced radiation dosage [7,8], reduced blood loss [9], and shorter hospital stay [9,10].

A critical factor in adopting new technology depends on the surgeon and staff overcoming the learning curve and becoming efficient with the use of the technology [1]. There is a learning curve for spinal robotics, shown to be approximately 3-30 cases to achieve proficiency based on the RAN spine surgery literature [11]. This initial learning curve is associated with the most rapid changes in associated operative times, fluoroscopy time, and screw accuracy, and some investigators have suggested this is followed by a relative plateau [11]. However, to the authors’ knowledge, literature reports have not focused beyond the initial learning curve. One report achieved a stable plateau of robot usage time after 20 cases, while the overall operative time continued to fluctuate [12]. Another report indicated that a plateau asymptote was reached after 25 navigated cases, with later patients requiring shorter operative and anesthesia times [13]. Furthermore, initial cases involved fewer complex surgeries, suggesting that as surgeons become more experienced with the RAN system, their confidence in the procedure increases, improving their ability to address more complex cases and allowing further exploration of the system’s versatility [13].

Although the minimum number of cases required to become proficient in RAN surgery is relatively low, it is possible that intraoperative outcomes, such as operative time and pedicle screw accuracy, are continuously refined and improved beyond the initial learning curve of RAN spine surgery. Refined outcomes beyond the initial learning curve have been studied in robot-assisted partial nephrectomy [14,15]. One report demonstrated continued outcome improvement beyond the initial learning curve and suggests that routine use of technology during cases normalizes utilization and may improve efficiency over time [14].

To assess RAN spine surgery outcomes with extended RAN experience, this study evaluated trends in perioperative outcomes and pedicle screw accuracy beyond the investigating surgeon’s 200th case, long after the purported initial learning curve was adequately overcome.

## Materials and methods

This retrospective institutional chart review was exempt from review by the Italian Ethics Committee. Informed consent was obtained from all participants as part of the institution’s standard practice. The study included 60 patients, all treated by the same attending surgeon, who met the following criteria: diagnosed with degenerative spine conditions and underwent transforaminal interbody fusion with lumbosacral pedicle screw placement with the use of robotic guidance and navigation (ExcelsiusGPS®, Globus Medical Inc., Audubon, PA). In this study, the robotic system operated on a preoperative computed tomography (CT) functional modality. 

To analyze outcome refinement of a fellowship-trained neurosurgeon with extensive robotic experience at a single site, 60 patients were sampled from the investigating surgeon’s total RAN case series and stratified into three groups based on case number. To the authors’ knowledge, a clear definition of extended RAN experience has yet to be defined in robotic-assisted spine surgery literature. Based on the investigating surgeon’s experience at the time of study, group 1 was established as a performance benchmark of 20 consecutive cases immediately leading up to the 200th case. Sequentially following group 1, groups 2 and 3 each included 20 additional consecutive cases, with an average of 15 cases between each group. This spacing allowed for better segmentation of groups and sampling of these 60 patients over a longer duration of the surgeon’s experience. Patients included in the study underwent the procedure between January 2020 and November 2021.

Included patients had the following data available: demographic data (including age, gender, body mass index (BMI), and diagnosis), operative data (including set-up time, screw insertion time, operative time, blood loss, and radiation time), CT scans, complications (screw-related and return to the operative room), and patient-reported outcomes (Oswestry Disability Index (ODI) and Visual Analog Pain Scores) available preoperatively and at least one month postoperatively.

In order to analyze RAN efficiency outcomes, operative time was segmented into three periods as follows: (1) Registration: positioning the robot in the operating room, attachment and activation of the registration fixture, and intraoperative 3D image acquisition of the surgical region, scan merging, and checks to ensure registration, (2) screw insertion time: period between positioning the robotic arm at the first pedicle to be instrumented to retraction of robotic arm following last screw insertion, and (3) total operative time: period between the first incision and closure.

The surgical technique described in this publication is similar to those detailed in previously published manuscripts by the same principal investigators [2,16]. Each patient received general anesthesia and was prepped and draped in standard fashion. Once the patient was placed on the table, the dynamic reference base was then placed by making a small incision and anchoring the base to the posterior superior iliac spine for registration. The fluoroscopy registration fixture was attached to the Ziehm Vision FD (Ziehm Imaging Inc., Nuremberg, Germany) and subsequently activated through a registration marker to ensure the robotic camera could verify its position. Fluoroscopy anteroposterior and lateral images were taken at each operative level to align the preoperative CT with the patient’s actual anatomy. Landmark checks were completed to ensure registration was calculated successfully.

A surgeon-controlled foot pedal positioned the robot arm to the most cephalad planned pedicle trajectory. Stab incisions were made on the skin using a scalpel. Navigated instruments were passed through the guide tube to maintain the planned trajectory, and screws were inserted with these instruments. This process was repeated for all screws. Rods were then placed, and locking caps were tightened in standard fashion. Intraoperative fluoroscopy images were obtained to verify the positioning of the screw and rod.

Postoperative CT scans were used to evaluate pedicle screw accuracy using the Gertzbein and Robbins System (GRS) [17]. In this scale, screws were graded as A (screw is completely within the pedicle), B (pedicle cortical breach of less than 2 mm), C (pedicle cortical breach of less than 4 mm), D (pedicle cortical breach of less than 6 mm), or E (pedicle cortical breach of more than 6 mm). Screws graded to have a less than 2 mm breach (Grade A or B) were considered clinically acceptable, while those screws with a greater than 2 mm breach (Grades C-E) were considered misplaced, as in other studies [2,18,19]. All images were calibrated, and measurements were taken using Surgimap® (Nemaris Inc., New York, NY, USA) using the line measurement tool.

Statistical analysis was performed using Excel (Microsoft Inc., Redmond, WA, USA) and IBM SPSS Statistics for Windows, Version 22.0 (Released 2013; IBM Corp., Armonk, NY, USA). Descriptive statistics were recorded as mean and standard deviation, or frequency and percentage, where applicable. Analysis of variance (ANOVA) was used to compare surgical parameters between RAN experience groups. Chi-square test was used to assess the association between fusion levels and accuracy across groups. Paired t-tests were performed to assess the differences in patient-reported outcomes from preoperative to postoperative follow-up. Any significant differences were reported. Statistical significance was set at p<0.05.

## Results

Patient demographics

A total of 60 patients underwent transforaminal interbody fusion with lumbosacral pedicle screw placement with the use of robotic guidance and navigation. Based on the RAN case number, patients were segmented to differentiate three robotic experience groups: extended experience groups 1, 2, and 3. The age ranged from 52 to 55 years and their BMI ranged from 23.4 to 25.1 across all groups. The most common diagnosis across all groups was degenerative disc disease. Groups had no significant differences in terms of age, sex, BMI, diagnosis, and operation level (Tables 1, 2).

**Table 1:** Patient and case demographics. BMI: body mass index.

Parameter	Group 1	Group 2	Group 3	p value
Number of patients	20	20	20	
Sex, n (%)				0.825
Male	12 (60%)	10 (50%)	11 (55%)	
Female	8 (40%)	10 (50%)	9 (45%)	
Age (years), mean	52±16	53±11	55±13	0.838
BMI (kg/m^2^), mean	24.3±3.2	25.1±3.2	23.4±3.0	0.266
Diagnosis, n (%)				0.344
Disc herniation	4 (20%)	3 (15%)	1 (5%)	
Spondylolisthesis	1 (5%)	1 (5%)	2 (10%)	
Degenerative disc disease	14 (70%)	12 (60%)	14 (70%)	
Stenosis	1 (5%)	2 (10%)	2 (10%)	
Anterolisthesis	0 (0%)	2 (10%)	1 (5%)	

**Table 2:** Case surgical characteristics. *Groups 1 and 2 statistically significant (p<0.05). †Groups 1 and 3 statistically significant (p<0.05). ‡Groups 2 and 3 statistically significant (p<0.05).

Parameter	Group 1	Group 2	Group 3	p value
Level of fusion, n (%)				0.929
Level 1	4 (20%)	5 (25%)	6 (30%)	
Level 2	13 (65%)	13 (65%)	11 (55%)	
Level 3	3 (15%)	2 (10%)	3 (15%)	
Estimated blood loss (cc), mean	54±27	45±29	50±18	
Radiation time (seconds), mean	33.6±40.2	82.2±37.2	72±13.2	
Operative time (minutes), mean^*, †^	175.9±58.2	135.83±23.9	121.5±32.3	
Registration time (minutes), mean^*, †,^^‡^	16.9±6.5	12.9±3.0	8.7±1.6	
Screw insertion time (minutes), mean^*, †, ‡^	14.9±3.5	10.9±2.0	8.4±2.7	

Operative data

**Figure 1:**
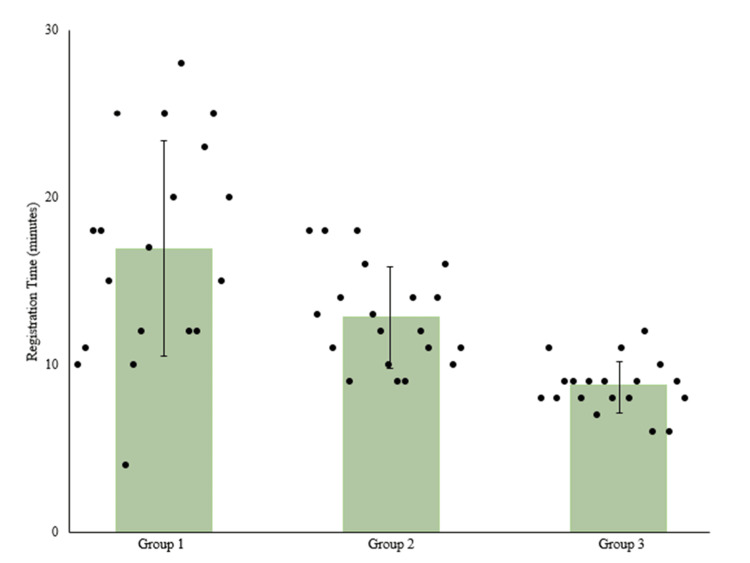
Scatter plot showing registration time, with bars representing the mean registration time.

The average registration time of group 1 (16.9±6.5 minutes) significantly decreased in group 2 (12.9±3.0 minutes) (p=0.014) and in group 3 (8.7±1.6 minutes) (p=0.008) (Figure 1).

**Figure 2:**
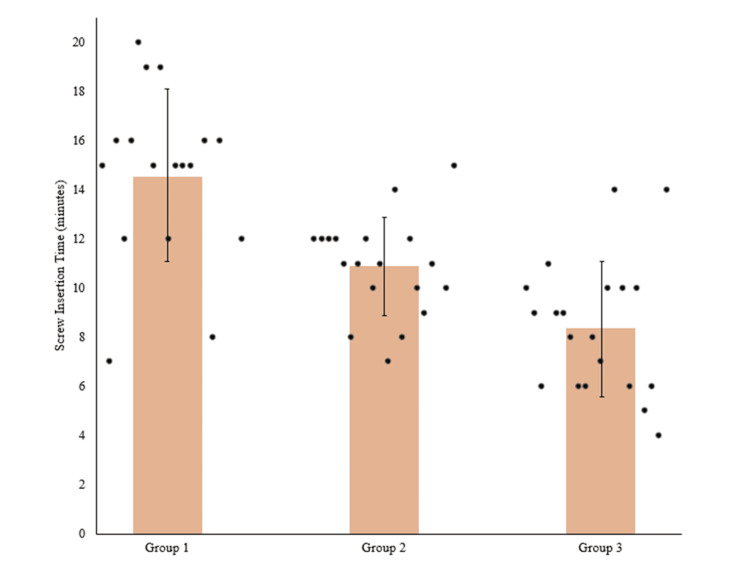
Scatter plot of screw insertion time, with bars representing the mean screw insertion time.

The average screw insertion time was 14.9±3.5 minutes in group 1 and significantly decreased to 10.9±2.0 minutes in group 2 (p=0.001), followed by a significant decrease to 8.4±2.7 minutes in group 3 (p=0.022) (Figure 2).

**Figure 3:**
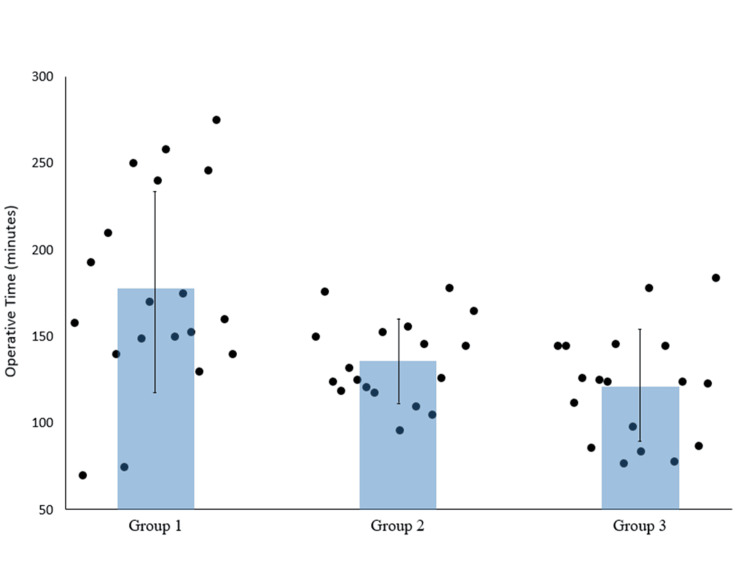
Scatter plot of total operative time, with bars representing the mean operative time.

Total operative time also significantly decreased from group 1 (175.9±58.2 minutes) to group 2 (135.8±23.9 minutes) (p=0.013), followed by a nonsignificant decrease in group 3 (121.5±32.3 minutes) (Figure 3).

All of the remaining surgical outcomes remained unchanged or statistically nonsignificant between the groups, including mean estimated blood loss (group 1: 54±27 cc, group 2: 45±29 cc, and group 3: 50±18 cc) and mean radiation time (group 1: 33.6±40.2 seconds, group 2: 82.2±37.2 seconds, and group 3: 72±13.2 seconds) (Table 2).

Accuracy

One to three levels were fused in each case, using a total of 290 pedicle screws with 108 in group 1, 84 in group 2, and 98 in group 3. Out of 108 screws placed in group 1, four surpassed the pedicle walls by ≥2 mm (Grades C-E), leading to a misplacement rate of 3.7% in group 1 (Table 3).

**Table 3:** Detailed summary of major pedicle screw breaches (Grades C–E).

Group	Case number	Vertebrae level (screw side)	Screw diameter (mm)	Screw length (mm)	Breach score	Directionality	Breach distance (mm)
1	3	L4 (left)	6.5	45	C	Lateral	2.6
1	5	L3 (left)	6.5	45	C	Lateral	3.4
1	16	L4 (left)	4.5	45	C	Medial	2.3
1	16	L5 (right)	4.5	45	C	Medial	2.2

**Figure 4:**
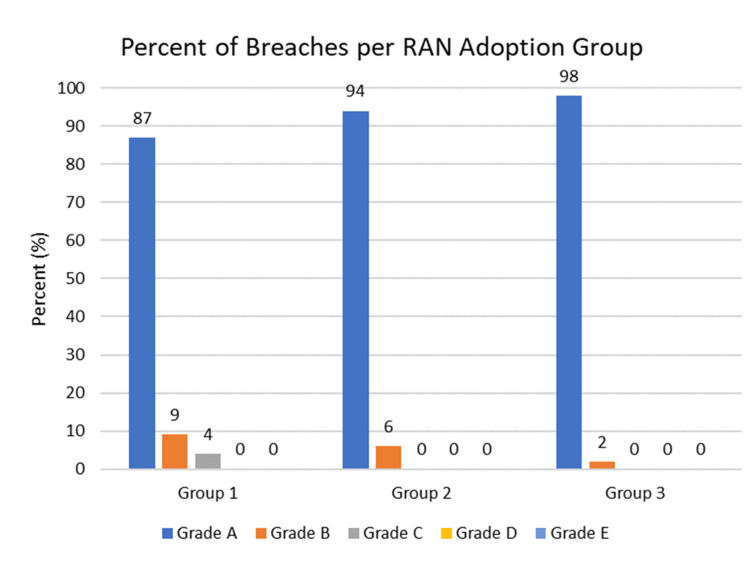
Rate of pedicle breach per breach grade in each RAN adoption group. RAN: robotic-assisted navigation.

**Figure 5:**
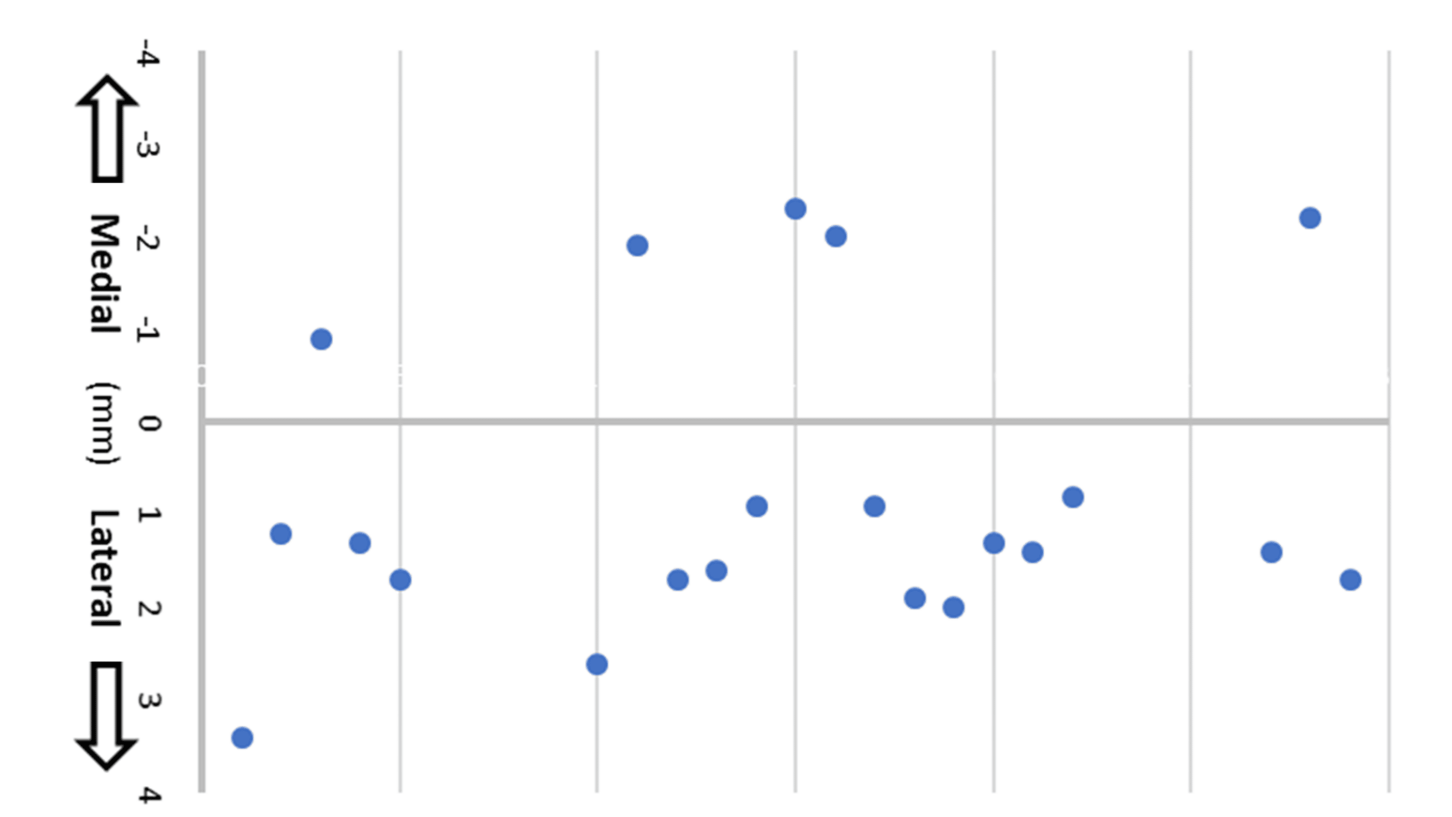
GRS of all breached screws (Grades B–E) showing more laterally shifted screws. GRS: Gertzbein and Robbins System.

With a total of 290 screws placed, the overall misplacement rate was 1.4%. The accuracy rate (Grade = A) significantly increased from 87% in group 1 to 94% in group 2 to 98% in group 3 (p=0.024) (Table 4, Figure 4). Of all breaches (Grades B-E), there was a higher rate of lateral screw misplacement (76.2%, 16/21) compared to medial misplacement (Figure 5).

**Table 4:** Detailed summary of GRS grading of all screws. GRS: Gertzbein and Robbins System. *Statistically significant (p<0.05).

			GRS					Total	p value
			Grade A	Grade B	Grade C	Grade D	Grade E		
Group	1	Count	94 (87.0%)	10 (9.3%)	4 (3.7%)	0 (0%)	0 (0%)	108	0.024*
		L2	0 (0%)	0 (0%)	0 (0%)	0 (0%)	0 (0%)	0 (0%)	
		L3	2 (2.1%)	3 (27.3%)	1 (25%)	0 (0%)	0 (0%)	6 (5.6%)	
		L4	30 (31.9%)	7 (63.6%)	2 (50%)	0 (0%)	0 (0%)	39 (36%)	
		L5	36 (38.3%)	0 (0%)	1 (25%)	0 (0%)	0 (0%)	37 (34.3%)	
		S1	26 (27.7%)	0 (0%)	0 (0%)	0 (0%)	0 (0%)	26 (24.1%)	
	2	Count	79 (94.0%)	5 (6.0%)	0 (0%)	0 (0%)	0 (0%)	84	
		L2	0 (0%)	0 (0%)	0 (0%)	0 (0%)	0 (0%)	0 (0%)	
		L3	10 (12.7%)	0 (0%)	0 (0%)	0 (0%)	0 (0%)	10 (12%)	
		L4	21 (26.6%)	5 (100%)	0 (0%)	0 (0%)	0 (0%)	26 (31%)	
		L5	30 (38.0%)	0 (0%)	0 (0%)	0 (0%)	0 (0%)	30 (35.7%)	
		S1	18 (22.8%)	0 (0%)	0 (0%)	0 (0%)	0 (0%)	18 (21.4%)	
	3	Count	98 (98.0%)	2 (2.0%)	0 (0%)	0 (0%)	0 (0%)	98	
		L2	4 (4.2%)	0 (0%)	0 (0%)	0 (0%)	0 (0%)	4 (4.0%)	
		L3	7 (7.3%)	1 (50%)	0 (0%)	0 (0%)	0 (0%)	8 (8.2%)	
		L4	30 (31.2%)	0 (0%)	0 (0%)	0 (0%)	0 (0%)	30 (30.6%)	
		L5	33 (34.4%)	1 (50%)	0 (0%)	0 (0%)	0 (0%)	34 (34.7%)	
		S1	22 (22.4%)	0 (0%)	0 (0%)	0 (0%)	0 (0%)	22 (22.4%)	
Total			269	17	4	0	0	290	

Clinical outcomes

Patient groups reported similar improvements in Visual Analog Scale (VAS) and ODI (%). Mean VAS scores of all groups significantly improved by 56.2% at one month postoperatively (7.3±1.2 to 3.2±1.0; p<0.0001). Mean ODI (%) scores of all groups also significantly improved by 49.1% at one month postoperatively (39.1±16.0 to 19.9±7.4; p<0.0001). There were no complications intraoperatively. There were no screw-related complications or returns to the operating room reported at one month postoperatively.

## Discussion

Based on previous reports, RAN spine surgery can be performed with consistently acceptable outcomes, including high pedicle screw placement accuracy [2,3,20], efficient intraoperative parameters [21,22], and minimal complications [5,23]. The overall surgical performance in our study was comparable to that in the literature. However, the majority of reports are limited to the initial RAN surgery experience, and to the authors’ knowledge, no studies directly report whether these outcomes continue to improve or truly plateau with extended experience after overcoming the initial learning curve. To further elucidate RAN spine surgery outcomes of an experienced RAN surgeon, we found that accuracy and surgical efficiency parameters were refined with RAN case experience beyond 200 cases. 

The learning curve of RAN spine surgery is most commonly measured by improvements in total operative time, pedicle screw accuracy, and screw placement time [11]. The accuracy of robotic screw placement has been studied extensively in the literature with results ranging from 85% to 100% of screws placed accurately and most rates fluctuating around 98% [17,24-27]. The present findings not only meet this standard but also continue to improve with more RAN case experience, well after overcoming the initial learning curve, with group 1 having 87% grade A, 9.3% grade B, and 3.7% grade C screws, followed by 94% grade A, 6% grade B, and 0% grade C screws in group 2 and 98% grade A, 2% grade B, and 0% grade C screws in group 3 (Figure 4).

Screw misplacement is reported between 1.1% and 28.2% in the literature; however, not every screw misplacement necessitates surgical revision [24]. In this study, group 1 had the highest misplacement rate of 3.7% (4/108 screws) and was the only group with major pedicle screw breaches (Grades C-E). The overall study misplacement rate was 1.4% (4/290 screws), with no surgical revisions necessary (Table 4). Based on patient-reported outcomes, including VAS and ODI scores, patients in this study reported immediate and significant symptom relief after RAN spine surgery. VAS improved by 56.2% at one month postoperatively and ODI improved by 49.1%. There were no intraoperative complications, and no screw-related complications or returns to the operating room were reported at one month.

The use of new technology always raises some important questions, such as skill development and assessment, teaching, and ease of translation into the operating room [12,28,29]. Devito et al. reported that of 682 procedures with 3,912 screw/guide wire insertions executed, 16.4% of screws were initiated under robotic guidance but were manually placed by the surgeon [18]. However, for the last 276 procedures, they found that the manual conversion rate decreased to 9.2%, and they attributed this improvement to the surgeon’s accumulated experience and structural and software improvements of the robotic system [12,18], thus suggesting that future plateaus are likely achieved. The refinement of pedicle screw accuracy found in our study for an experienced RAN user is likely attributed to the surgeon’s increased familiarity and trust in the imaging and robotic system and screw planning software. 

It is critical to emphasize the importance of spinal anatomy mastery and fellowship training with competency in freehand or fluoroscopically confirmed screw placement before developing a proficiency in navigation and robotics [30]. Moreover, placement of the screw within the boundaries of the pedicle is dependent on the accuracy of the screw to the plan developed by the surgeon [16]. From the general trend in literature, it appears that the majority of robotically placed screws are lateral [20,27,31]. Ringel et al. found that when placing free hand screws, lateral misplacements represent 30% as compared to 70% of all misplacements when using robotics [27]. This aligns with our observation that 76% of all misplaced screws (Grades B-E) were lateral (Figure 5). One study suggests to avoid encroaching into the spinal canal, there is an unconscious tendency to plan trajectories more laterally than necessary [20]. Zhou et al. attributed poor drilling and skiving caused by irregular bony surfaces, steep entry point angles, and pressure from soft tissues to deviation of robotic-assisted navigated screws from planned trajectories [32,33]. Nevertheless, familiarity with RAN techniques and instruments, such as a high-speed drill, can substantially improve pedicle screw accuracy [32,34].

One major barrier to the adoption of RAN surgery is the concern about registration time and added operative time to the procedure [35]. In this study, registration time included robot positioning in the operating room, attachment and activation of the registration fixture, intraoperative 3D image acquisition of the surgical region, scan merging, and anatomy landmark checks. The seemingly long registration times reported across all groups in this study compared to 9.1±2.0 minutes reported in the literature [36] can be attributed to more items being measured in the registration time calculation, such as robot positioning in the operating room and attachment and activation of the registration fixture. While the registration process is variable and dependent on different factors, such as experience of staff handling intraoperative image acquisition, patient’s spinal anatomy, obesity, bone quality, and vertebral level [36], this study reported a significant decrease in registration time across groups (p<0.05) (Figure 1). In addition, refined screw insertion time was achieved. Literature reports time per robotically placed screw at 3.6-5.5 minutes after the initial learning curve [2,11,31]. Our study reports screw insertion time below that at 2.7±1.2 minutes per screw (14.6±3.5 minutes per case) reported in group 1, with improvements to 2.1 ± 0.6 per screw (10.9±2.0 minutes per case) in group 2 and 1.5±0.3 per screw (8.4±2.7 minutes per case) in group 3. 

Both registration and screw insertion time refinement impacted the total operative time reported. Schroder et al. reported an average total operative time of single-level minimally invasive transforaminal or posterior lumbar interbody fusion of 161±50 minutes [24]. This study reports comparable total operative times and a significant decrease across groups (p<0.05) (Figure 3).

Literature supports that general proficiency is reached after the initial learning curve; however, the present results suggest that additional outcome refinement is achievable with extended or “expert” level experience. Ultimately, the incremental improvement of outcomes achieved in the present study is attributable to a large-scale RAN implementation, which was reflected in the surgeon’s and operating staff’s familiarity and experience with the image and robotic navigation system and procedural workflow finetuning. 

Limitations

It is important to note that the results of this study should be interpreted within the context of a single location and derived from a single surgeon’s experience, which impacts the generalizability of the results. The strengths of this study include the addition of registration and screw placement time data capture and the uniqueness of the study concept. Limitations of this study include the retrospective study design with relatively small and subjective patient samples based on the investigating surgeons’ experience at the time of the study and lack of long-term clinical outcomes. It is important to note that extended RAN experience is understudied, and this report acts an initial report, as future studies with different robotic devices, surgical workflows, and robotic techniques involving factors such as obesity and case complexity are needed to further define and elucidate the measurability of extended RAN experience. 

## Conclusions

This study addresses a single surgeon’s extended RAN spine surgery experience with respect to pedicle screw accuracy and intraoperative efficiencies, such as registration, screw placement, and total operative time. While RAN spine surgery can consistently be performed with high accuracy and acceptable intraoperative outcomes even during an initial learning curve period, this study demonstrated continual refinement well after the initial learning curve was overcome with extended RAN experience beyond 200 cases. All patient groups reported significant postoperative improvements in terms of pain and function at one month, with no intraoperative complications or returns to the operating room.
